# Health-related quality of life and associated factors among individuals with cancer in Laos

**DOI:** 10.3389/fpubh.2026.1941268

**Published:** 2026-10-09

**Authors:** Nithaya Xatakoune, Lena J. Lee, Hohyun Seong, Jennifer J. Barb, Aminata J. Sinyan, Li Yang, Ran Lee

**Affiliations:** 1 Champasak College of Health Sciences, Pakse, Lao People's Democratic Republic; 2 Translational Biobehavioral and Health Promotion (TBHP) Branch, National Institutes of Health (NIH) Clinical Center, Gwangju, MD, United States; 3 College of Nursing, Chonnam National University, Uijeongbu-si, Republic of Korea; 4 Johns Hopkins Bloomberg School of Public Health, Baltimore, MD, United States; 5 Kennedy Krieger Institute, Baltimore, MD, United States; 6 Department of Nursing, Kyungmin University, Uijeongbu-si, Republic of Korea

**Keywords:** health inequities, Laos, LMICs, neoplasms, quality of life

## Abstract

**Introduction:**

Being diagnosed with cancer has diverse consequences that can influence patients’ health-related quality of life (HRQoL). Understanding HRQoL and its associated factors among cancer patients, especially in low- and middle-income countries, is critical to focus on early detection and enhance cancer treatment strategies. In Laos, however, there is a scarcity of information. The study aimed to assess HRQoL in individuals with cancer in Laos and its association with sociodemographic and clinical factors.

**Materials and methods:**

Data were obtained from 193 patients with diverse cancers in Laos. HRQoL was assessed using a 30-item of European Organization for Research and Treatment of Cancer quality of life questionnaire-C30 (EORTC QLQ–C30), including nine subscales: global health status/quality of life (GQoL), functioning (physical, role, emotional, cognitive, social), and symptoms (pain, fatigue, nausea/vomiting). Predetermined thresholds indicating clinical importance were applied to identify patients exhibiting functional impairment and symptoms at a level meriting concern (clinical case). Controlling for age, sex and cancer stage, multiple linear and logistic regression models were used to identify factors associated with subscales of EORTC QLQ-C30.

**Results:**

More than three-quarters of the patients reported functioning at levels corresponding to clinical cases (54–93%) and experiencing pain (82%), nausea/vomiting (83%), and financial difficulties (87%). Higher educational attainment and receipt of cancer treatment were associated with better GQoL. Comorbid conditions were consistently associated with poorer functioning and greater symptom burden, including fatigue, dyspnea, constipation, and diarrhea.

**Conclusion:**

Cancer patients in Laos experience a substantial burden of clinically important functional impairments, symptoms, and financial difficulties. Sociodemographic and clinical factors, particularly education, treatment modality, and comorbid conditions, were associated with HRQoL outcomes. Participants who had not received treatment generally experienced greater functional impairment and symptom burden, underscoring the importance of timely access to cancer care and supportive services. These findings support the need for targeted supportive care interventions and symptom management strategies to improve the wellbeing of cancer patients in Laos.

## 1 Introduction

Cancer is a major public health concern worldwide and remains one of the leading causes of morbidity and mortality. The burden of cancer is increasing in low- and middle-income countries (LMICs) due to demographic changes, population aging, and epidemiological transition (1). The Lao People’s Democratic Republic (Lao PDR) is undergoing a demographic and epidemiological transition characterized by the coexistence of communicable diseases and undernutrition with a growing burden of non-communicable diseases, including cancer (1, 2). According to Global Cancer Observatory (GLOBOCAN) estimates, Laos recorded 9,101 new cancer cases and 6,215 cancer-related deaths in 2022, and the cancer burden is projected to increase substantially in the coming decade (2–4).

Beyond its impact on morbidity and mortality, cancer has profound consequences for patients’ health-related quality of life (HRQoL). HRQoL is a multidimensional concept encompassing physical, emotional, cognitive, social, and role functioning, as well as disease- and treatment-related symptoms (5). Cancer patients commonly experience pain, fatigue, nausea, vomiting, insomnia, and psychological distress, which may interfere with daily activities and reduce overall wellbeing (5, 6). Consequently, HRQoL has become an important patient-reported outcome and an essential component of comprehensive cancer care (7).

Assessment of HRQoL is particularly important in LMICs, such as Lao PDR, where cancer patients often face delayed diagnosis, limited access to specialized treatment and supportive care services, and substantial financial burdens associated with cancer care (8, 9). In Lao PDR, financial barriers to cancer care remain substantial despite the implementation of the National Health Insurance scheme. Previous studies have shown that although many households are covered under the insurance scheme, a considerable proportion continue to incur high out-of-pocket healthcare expenditures, particularly those with family members affected by chronic diseases (10, 11). In addition, patients requiring complex cancer treatment have often sought care in neighboring countries, particularly Thailand, resulting in substantial travel and out-of-pocket expenses (12). In these settings, HRQoL assessment can provide valuable information beyond traditional clinical outcomes by identifying unmet patient needs, informing supportive care interventions, guiding healthcare policy, and facilitating the efficient allocation of limited healthcare resources (8, 9, 11).

Studies conducted in Southeast Asia have reported substantial impairments in HRQoL among cancer patients and survivors, with symptom burden and socioeconomic disadvantage contributing significantly to poorer outcomes (13). In addition, previous studies have shown that HRQoL among cancer patients is influenced by both sociodemographic and clinical factors. Characteristics such as education, income, marital status, and employment, as well as clinical factors including cancer stage, treatment modality, and comorbid conditions, have been associated with variations in HRQoL outcomes (14, 15). Despite the growing cancer burden in Lao PDR, evidence regarding HRQoL and its associated factors among cancer patients remains scarce. Routine health information systems are essential for monitoring disease burden, evaluating health services, and informing cancer control policies. However, Lao PDR currently does not have a population-based cancer registry, and its health information system faces several challenges, including inconsistencies in data collection across healthcare facilities and infrequent data updates (16, 17). These limitations hinder accurate assessment of the national cancer burden and constrain evidence-based planning for cancer prevention, treatment, and supportive care. Consequently, healthcare providers and policymakers have limited evidence to identify patients with unmet supportive care needs, prioritize symptom management, allocate healthcare resources efficiently, and develop evidence-based cancer control policies. Generating HRQoL data is therefore essential to support patient-centered cancer care and inform clinical practice and health policy in Lao PDR. Accordingly, this study aimed to assess HRQoL among adult cancer patients in Laos using the European Organization for Research and Treatment of Cancer Quality of Life Questionnaire-Core 30 (EORTC QLQ-C30) and to examine sociodemographic and clinical factors associated with HRQoL.

## 2 Materials and methods

### 2.1 Study design, participants, and setting

This study employed a cross-sectional survey design to investigate HRQoL among patients diagnosed with cancer for at least 3 months at three major tertiary referral hospitals Vientiane, Lao PDR: the National Cancer Center, Settthathirath Hospital, and Mahosot Hospital. Data collection was carried out between February and March 2022. Participants were eligible if they (1) were aged 18 years or older, and (2) had been diagnosed with cancer for at least 3 months. Patients were excluded if they (1) had other severe chronic conditions that could substantially influence HRQoL independently of cancer, such as diabetes mellitus or heart disease, (2) refused to participate, or (3) had cognitive or mental disorders. Patients with stable comorbid conditions, including hypertension and anemia, were eligible for participation. The required sample size was proportionally allocated across the three hospitals based on their average monthly oncology patient volume. Of the 223 participants who enrolled, 96 (43.0%) were recruited from the National Cancer Center, 65 (29.2%) from Setthathirath Hospital, and 62 (27.8%) from Mahosot Hospital.

### 2.2 Data collection

Participants were recruited using proportionate quota sampling with consecutive recruitment. During the study period, a total of six trained oncology research nurses (two nurses at each hospital) conducted data collection. The research nurses received standardized training on eligibility screening, informed consent procedures, questionnaire administration, and procedures for assisting participants with limited literacy. They screened eligible patients attending outpatient oncology clinics or admitted to inpatient oncology wards during data collection hours until the predetermined sample quota for each hospital was achieved. They explained the study objectives and procedures to eligible participants and obtained written informed consent before enrollment. Participants then completed a self-administered paper questionnaire, which required approximately 15–20 min to complete. For participants who required assistance because of limited literacy, trained research nurses read the questionnaire items aloud and recorded participants’ responses without influencing their answers. Completed questionnaires were reviewed for completeness at the time of collection, and data were checked for completeness and consistency before analysis. Of the 223 patients who agreed to participate, 193 were included in the final analysis after excluding 30 individuals with incomplete responses to the HRQoL questionnaire. The excluded questionnaires contained missing items within key functional and symptom scales that exceeded the level permitted under the EORTC QLQ-C30 scoring guidelines, preventing valid scale score calculation. Baseline demographic and clinical information for these 30 participants was unavailable because of incomplete baseline administrative records.

### 2.3 Measures

#### 2.3.1 General characteristics

Socio-demographic characteristics included age, sex, marital status, education, employment, religion, and household income. Household income was converted from Lao kip (LAK) to US dollars (USD) to facilitate international comparisons. Clinical characteristics included type of cancer, stage of cancer, type of cancer treatment, and comorbid conditions. Type of cancer treatment was defined based on treatment received before or being received at the time of the survey and was categorized as no treatment, single-modality treatment (surgery alone, chemotherapy alone, or radiotherapy alone), or combination therapy. Patients who had not received any cancer treatment before or at the time of the survey, including those awaiting treatment, were classified in the no-treatment group.

#### 2.3.2 The EORTC QLQ-C30

The EORTC QLQ-C30 (version 3.0) is a self-administered, cancer-specific instrument developed to assess quality of life among patients with cancer, regardless of cancer type (6). All questionnaires used in this study were translated into the Lao language following the procedures described by Hilton and Skrutkowski (18). First, the questionnaires were translated from English into Lao by the researcher. Second, back-translation into English was performed by bilingual experts who were not involved in the initial translation and had not reviewed the original English versions, thereby minimizing potential translation bias. Third, the researcher and bilingual experts compared the original English versions with the back-translated versions to assess linguistic equivalence, conceptual consistency, and cultural appropriateness. Any discrepancies or culturally inappropriate wording were discussed with the research advisor and experts, and revisions were made until consensus was reached.

The questionnaire consists of 30 items grouped into nine multi-item scales: physical functioning (5 items), role functioning (2 items), emotional functioning (4 items), cognitive functioning (2 items), social functioning (2 items), fatigue (3 items), nausea and vomiting (2 items), pain (2 items), and global health status/quality of life (GQoL) (2 items). In addition, six single-item scales assess dyspnea, sleep disturbance, appetite loss, constipation, diarrhea, and financial difficulties. All items, except the global health status/quality of life scale, are rated on a 4-point Likert scale ranging from 1 (*not at all*) to 4 (*very much*). The global health status/quality of life scale is rated on a 7-point scale ranging from 1 (*very poor*) to 7 (*excellent*). Raw scores were transformed linearly to a 0–100 scale according to the EORTC QLQ-C30 scoring manual (6). Higher scores on the functioning and global health status scales indicate better functioning and quality of life, whereas higher scores on the symptom scales indicate greater symptom burden. Cronbach’s alpha coefficients ranged from 0.71 to 0.80, indicating acceptable to good internal consistency.

Thresholds for Clinical Importance (TCIs) established by Giesinger et al. (19) were used to identify patients with clinically important impairments and symptoms. Participants were classified as meeting or not meeting the threshold for clinical importance for each QLQ-C30 domain. The TCI cutoffs for the functioning scales were 83 for physical functioning, 58 for role functioning, 58 for social functioning, 71 for emotional functioning, and 75 for cognitive functioning. The TCI cutoffs for the symptom scales were 39 for fatigue, 25 for pain, 8 for nausea/vomiting, 50 for sleep disturbance, 17 for dyspnea, 50 for appetite loss, 50 for constipation, 17 for diarrhea, and 17 for financial difficulties.

### 2.4 Statistical analysis

Means, standard deviation, frequencies, and percentage were reported for all variables. Data distribution and normality were checked using the Shapiro–Wilk tests and normal QQ plots for all continuous variables. Bivariate analyses were conducted to examine associations between HRQoL outcomes and sociodemographic and clinical characteristics. Parametric tests (Pearson correlation, independent-samples t test, and one-way analysis of variance [ANOVA]) or nonparametric tests (Spearman correlation, Wilcoxon rank-sum test, and Kruskal-Wallis test) were used as appropriate. As the primary HRQoL outcome measured by EORTC QLQ-C30, GQoL was analyzed as a continuous variable, whereas the functioning and symptom variables were categorized based on the TCIs. Logistic regression models were used as the primary analyses for the functioning and symptom outcomes to evaluate clinically important HRQoL problems and facilitate clinical interpretation. Independent variables with a *p* value <0.10 in the bivariate analyses were additionally entered into the multiple linear or logistic regression models. When no additional variables met the screening criterion a covariate-only model including age, sex, and cancer stage was fitted. As sensitivity analyses, multiple linear regression analyses were conducted using the original continuous scores for the functioning and symptom scales, with the same predictors included in the corresponding primary logistic regression models. These analyses were conducted to assess the robustness of the findings when HRQoL outcomes were analyzed as continuous rather than dichotomized variables. Because the analyses were exploratory, no formal adjustment for multiple comparisons was performed. Model assumptions were assessed using residual and normality probability plots to evaluate normality and homoscedasticity. Transformations were performed when necessary. Multicollinearity was assessed using tolerance and the variance inflation factor (VIF) (13). All the statistical analyses were performed utilizing the IBM SPSS software package version 29.0.

## 3 Results

### 3.1 Sample characteristics

A total of 193 patients with cancer participated in the study. The sociodemographic and clinical characteristics of the study participants are shown in Table 1. The mean age was 53.6 years (SD = 11.7), and 61.1% were female. Most participants were married (93.8%) and Buddhist (91.7%). Nearly half had completed primary education (49.7%), and the mean monthly household income was USD 391.5 (SD = 162.6). Most patients had advanced disease, with 38.0% diagnosed at Stage III and 38.6% at Stage IV. Colorectal cancer (29.5%) and breast cancer (18.1%) were the most common cancer types. Over half of the participants had at least one comorbid condition (51.3%), primarily hypertension (44.0%). The most common treatment modality was surgery combined with chemotherapy (28.5%).

**Table 1:** Descriptive characteristics (*N* = 193).

Characteristics	*n* (%) Mean ± SD, range
Gender	
Male	75 (38.9)
Female	118 (61.1)
Age (years)	53.59 ± 11.66, 18–85
Marital status	
Single	11 (5.7)
Married	181 (93.8)
Widowed	1 (0.5)
Religion	
Buddhism	177 (91.7)
Christian	1 (0.5)
Others	15 (7.8)
Education level	
Primary school	94 (49.7)
High school	48 (25.4)
Bachelor degree	21 (11.1)
Graduate degree	26 (13.8)
Employment	
Housewife	66 (34.2)
Farmer	38 (19.7)
Merchant	25 (13.0)
Government employee	21 (10.8)
Private sectors employee	17 (8.8)
Retired	10 (5.2)
Student	3 (1.6)
Others	13 (6.7)
Household income (monthly, USD)	391.50 ± 162.59, 200–1,200
Stage of cancer	
Stage I	8 (4.3)
Stage II	35 (19.1)
Stage III	70 (38.0)
Stage IV	71 (38.6)
Type of cancer	
Breast cancer	35 (18.1)
Lung cancer	14 (7.3)
Liver cancer	16 (8.3)
Colorectal cancer	57 (29.5)
Prostate cancer	5 (2.6)
Stomach cancer	17 (8.8)
Cervical cancer	24 (12.4)
Others	25 (13.0)
Comorbidity	
None	94 (48.7)
Hypertension	85 (44.0)
Anemia	6 (3.2)
Others	8 (4.1)
Type of cancer treatment	
None	50 (25.9)
Surgery alone	33 (17.1)
Chemotherapy alone	37 (19.2)
Radiotherapy alone	6 (3.1)
Surgery and chemotherapy	55 (28.5)
Chemotherapy and radiotherapy	10 (5.2)
Surgery and radiotherapy	2 (1.0)

Numbers may not sum to total due to missing data.

SD: Standard Deviation.

### 3.2 Health-related quality of life (HRQoL)

HRQoL subscale scores and the prevalence of clinically important functional impairments and symptoms are presented in Table 2. The mean GQoL score was 56.7 ± 21.1. Because no established TCI is available for this scale, only descriptive statistics are presented. Based on the EORTC QLQ-C30 thresholds for clinical importance, a high proportion of participants reported clinically important functional impairments and symptoms (Table 2). Among the functional domains, emotional functioning was the most affected, with 93.3% of patients meeting the TCIs, followed by physical functioning (90.2%), cognitive functioning (82.4%), social functioning (79.3%), and role functioning (53.9%). Regarding symptom scales, clinically important nausea/vomiting (82.9%) and pain (82.4%) were the most frequently reported symptoms, followed by fatigue (69.4%). Among the single-item scales, financial difficulties were the most prevalent concern, affecting 87.0% of participants. Clinically important dyspnea was reported by 69.4% of patients, while approximately half experienced clinically important diarrhea (50.3%), loss of appetite (49.7%), and sleep disturbances (49.2%). Constipation was reported by 26.9% of participants.

**Table 2:** HRQoL Subscale scores and prevalence of clinically important problems/symptoms (positive cases) and those without (negative cases; *N* = 193).

Subscales	Mean ± SD	Positive case^a^			Negative case^b^		
		Mean ± SD	*n*	%	Mean ± SD	*n*	%
Global health status/quality of life	56.73 ± 21.12	–			–		
Functional subscales							
Physical functioning	58.13 ± 19.29	54.83 ± 17.32	174	90.2	88.42 ± 3.75	19	9.8
Role functioning	56.99 ± 27.19	35.98 ± 14.46	104	53.9	81.65 ± 15.08	89	46.1
Emotional functioning	45.03 ± 21.04	42.50 ± 19.38	180	93.3	80.13 ± 7.25	13	6.7
Cognitive functioning	57.34 ± 22.99	49.58 ± 16.87	159	82.4	93.63 ± 8.22	34	17.6
Social functioning	33.07 ± 27.66	22.98 ± 21.12	153	79.3	71.67 ± 10.13	40	20.7
Symptoms							
Pain	46.71 ± 25.81	55.24 ± 19.50	159	82.4	6.86 ± 8.33	34	17.6
Fatigue	52.61 ± 20.24	62.27 ± 16.46	134	69.4	30.70 ± 5.59	59	30.6
Nausea and vomiting	38.16 ± 27.04	46.04 ± 22.78	160	82.9	0	33	17.1
Single-item scales							
Dyspnea	38.68 ± 32.81	55.72 ± 24.45	134	69.4	0	59	30.6
Sleep disturbance	51.46 ± 29.64	77.54 ± 15.71	95	49.2	26.19 ± 13.75	98	50.8
Appetite loss	52.84 ± 31.25	80.21 ± 16.46	96	49.7	25.77 ± 14.03	97	50.3
Constipation	30.91 ± 33.41	78.85 ± 16.21	52	26.9	13.24 ± 16.37	141	73.1
Diarrhea	30.22 ± 36.02	60.14 ± 27.90	97	50.3	0	96	49.7
Financial difficulties	71.67 ± 36.69	82.43 ± 25.77	168	87.0	0	25	13.0

^a^*Positive case*: Indicates individuals who meet the threshold for clinical importance for this quality of life measure; ^b^*Negative case*: Indicates individuals who do not meet this threshold. Thresholds for clinical importance are not available for the Global Health Status/QoL scale. SD: Standard Deviation; EORTC QLQ-C30: European Organization for Research and Treatment of Cancer Quality of Life Questionnaire-Core 30-items.

### 3.3 Associations between factors and HRQoL

Figure 1 presents the forest plots of the final multiple linear regression model for GQoL, and the final logistic regression models for each of the other outcomes. Sociodemographic variables included age, sex (female vs. male), marital status (single/divorced/widowed vs. married), education level (primary or high school vs. ≥ bachelor’s degree), and household income (19). Clinical variables included cancer stage (stage I/II vs. stage III/IV), treatment modality (no treatment, single-modality treatment [surgery alone, chemotherapy alone, or radiotherapy alone], and combination therapy), and comorbid conditions (no vs. yes). Household income was natural log transformed before inclusion in the regression analyses because of its skewed distribution. Accordingly, estimates for household income were reported per doubling of income to facilitate interpretation. Age, sex, and cancer stage were controlled for in all models. Adjusted regression results are summarized below for statistically significant factors other than the *a priori* covariates (age, sex, and cancer stage); complete adjusted estimates for all variables are presented in Supplementary Table 1. Overall, sensitivity analyses using the original continuous scores yielded findings comparable to those from the primary logistic regression analyses (Supplementary Table 2).

**Figure 1:**
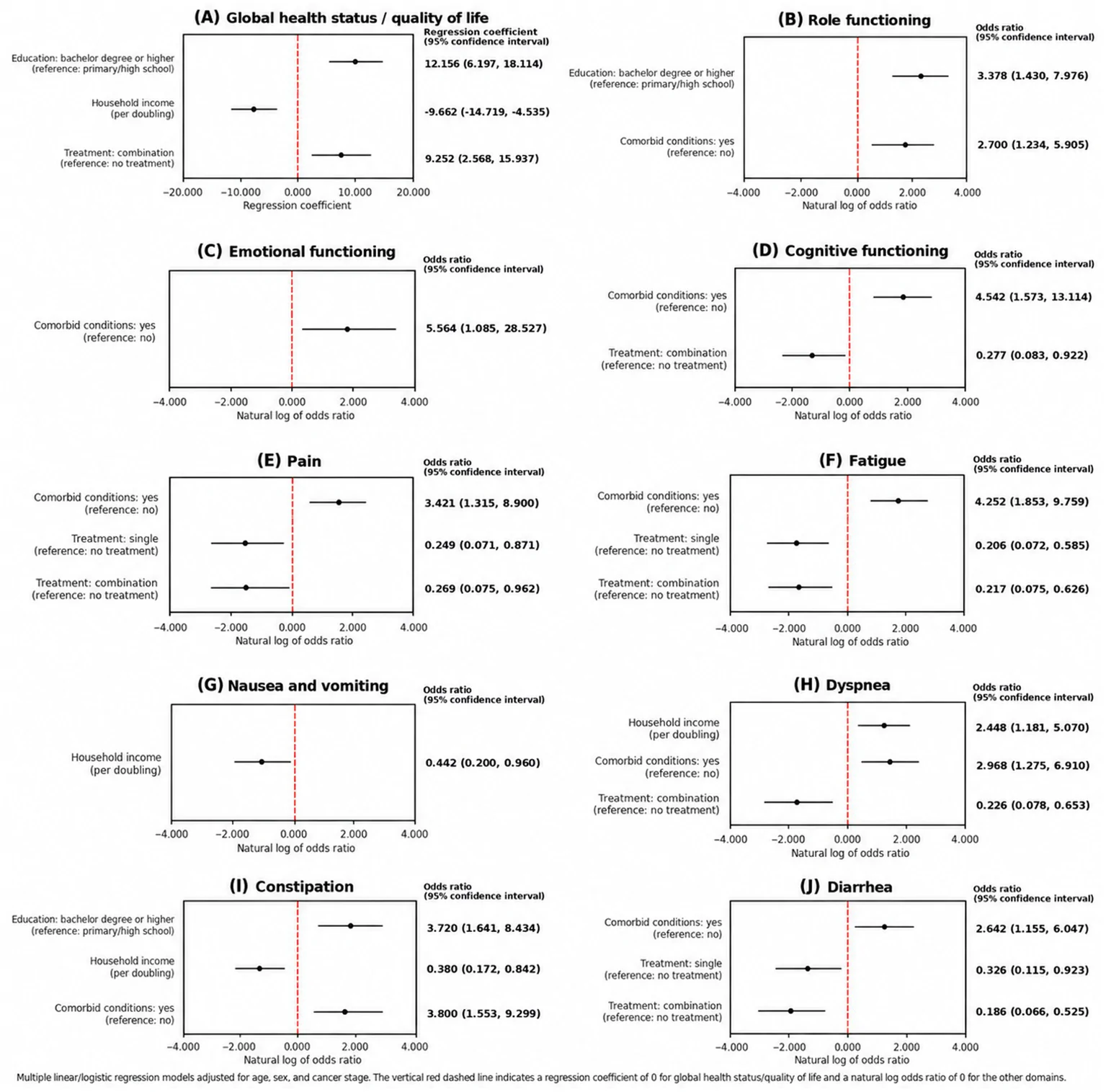
Forest plots of factors associated with the global status/quality of life, and clinically important functional impairments and symptoms. **(A)** Global health status/quality of life, **(B)** Role Functioning, **(C)** Emotional Functioning, **(D)** Cognitive Functioning, **(E)** Pain, **(F)** Fatigue, **(G)** Nausea and Vomiting, **(H)** Dyspnea, **(I)** Constipation, **(J)** Diarrhea (CI = confidence interval; OR = odds ratio).

#### 3.3.1 GQoL

Three variables, education, income, and treatment, were included in the multiple linear regression model for GQoL. Participants with a reported bachelor’s degree or higher had significantly better GQoL than those with a primary or high school education (*B* = 12.156, 95% confidential interval [CI; 6.197, 18.114], *p* < 0.001). A doubling of income was associated with a 9.662-point lower GQoL score (95% CI [−14.719, −4.535], *p* < 0.001), after adjustment for other variables in the model. Regarding treatment, participants receiving combined treatment modalities reported higher GQoL scores than those who had not received treatment (*B* = 9.252, 95% CI [2.568, 15.937], *p* = 0.007).

#### 3.3.2 Physical functioning

Two factors (comorbidity and treatment) met the bivariate analyses criteria and were included in a multiple logistic regression model predicting physical functioning. No additional factor met the criterion for inclusion in the final regression model.

#### 3.3.3 Role functioning

Four variables (education, income, comorbidity, and treatment) were included in the multiple logistic regression model for role functioning. Participants with a bachelor’s degree or higher were more likely to report impaired role functioning compared with those with a primary or high school education (OR = 3.378, 95% CI [1.430, 7.976], *p* = 0.005). In addition, participants with comorbid conditions were more likely to report impaired role functioning than those without comorbid conditions (OR = 2.700, 95% CI [1.234, 5.905], *p* = 0.013).

#### 3.3.4 Emotional functioning

Comorbidity was the only factor to meet the bivariate association criteria with emotional functioning and was included in a final regression model. Patients with comorbid conditions had significantly higher odds of clinically important emotional impairment than those without comorbidities (OR = 5.564, 95% CI [1.085, 28.527], *p* = 0.040).

#### 3.3.5 Cognitive functioning

Three factors (marital status, comorbidity, and treatment) met the bivariate analyses criteria and were included in a multiple logistic regression model predicting cognitive functioning. Participants with comorbid conditions were more likely to report cognitive impairment than those without comorbidities (OR = 4.542, 95% CI [1.573, 13.114], *p* = 0.005). Participants who received combination therapy had significantly lower odds of reporting clinically important cognitive impairment than those who received no treatment (OR = 0.277, 95% CI [0.083–0.922], *p* = 0.036).

#### 3.3.6 Social functioning

No additional factors met the bivariate analysis criteria for inclusion; therefore, a covariate-only logistic regression model including age, sex, and cancer stage was fitted for social functioning.

#### 3.3.7 Pain

Two factors (comorbidity and treatment) met the bivariate screening criteria and were entered into the multiple logistic regression model for pain. Patients with comorbid conditions had significantly higher odds of reporting clinically important pain than those without comorbidities (OR = 3.421, 95% CI [1.315–8.900], *p* = 0.012). Compared with participants who received no treatment, those who received single-modality treatment or combination therapy had significantly lower odds of reporting clinically important pain (OR = 0.249, 95% CI [0.071–0.871], *p* = 0.030; OR = 0.269, 95% CI [0.075–0.962], *p* = 0.044, respectively).

#### 3.3.8 Fatigue

Two factors (comorbidity and treatment) met the bivariate analyses criteria and were included in a multiple logistic regression model predicting fatigue. Participants with comorbid conditions had significantly higher odds of reporting clinically important fatigue than those without comorbid conditions (OR = 4.252, 95% CI [1.853–9.759], *p* < 0.001). Compared with participants who received no treatment, those who received single-modality treatment or combination therapy had significantly lower odds of reporting clinically important fatigue (OR = 0.206, 95% CI [0.072–0.585], *p* = 0.003; OR = 0.217, 95% CI [0.075–0.626], *p* = 0.005, respectively).

#### 3.3.9 Nausea/vomiting

Three factors (education, household income, and comorbidity) met the bivariate analyses criteria and were included in a multiple logistic regression model predicting nausea/vomiting. A doubling of income was associated with 56% lower odds of clinically important nausea/vomiting (OR = 0.442, 95% CI [0.200–0.960], *p* = 0.039), after adjustment for other variables in the model.

#### 3.3.10 Dyspnea

Four variables (marital status, income, comorbidity, and treatment) were included in the multiple logistic regression model for dyspnea. A doubling of income was associated with 2.448 times higher odds of clinically important dyspnea (OR = 2.448, 95% CI [1.181, 5.070], *p* = 016), after adjustment for other variables in the model. In addition, participants with comorbid conditions had significantly higher odds of reporting clinically important dyspnea than those without comorbid conditions (OR = 2.968, 95% CI [1.275–6.910], *p* = 0.012). Participants who received combination therapy had significantly lower odds of clinically important dyspnea than those who received no treatment (OR = 0.226, 95% CI [0.078–0.653], *p* = 0.006).

#### 3.3.11 Sleep disturbance

Marital status was the only additional factor that met the bivariate screening criterion in the final model for sleep disturbance along with age, sex, and cancer stage.

#### 3.3.12 Appetite loss

Household income was the only additional factor that met the bivariate screening criterion in the final model for appetite loss along with age, sex, and cancer stage.

#### 3.3.13 Constipation

Three factors (education, household income, and comorbidity) met the bivariate analyses criteria and were included in a multiple logistic regression model predicting constipation. Participants with at least a bachelor’s degree had higher odds of reporting clinically important constipation compared with those with a primary/high school education (OR = 3.720, 95% CI [1.641, 8.434], *p* = 0.002). A doubling of income was associated with 62% lower odds of clinically important constipation (OR = 0.380, 95% CI [0.172–0.842], *p* = 0.017), after adjustment for other variables in the model. In addition, participants with comorbid conditions had significantly higher odds of reporting clinically important constipation than those without comorbid conditions (OR = 3.800, 95% CI [1.553, 9.299], *p* = 0.003).

#### 3.3.14 Diarrhea

Three factors (education, comorbidity, and treatment) met the bivariate screening criteria and were entered into the multiple logistic regression model for diarrhea. Participants with comorbid conditions had higher odds of reporting clinically important diarrhea compared with those without comorbid conditions (OR = 2.642, 95% CI [1.155, 6.047], *p* = 0.021). Compared with participants who received no treatment, those who received single-modality treatment or combination therapy had significantly lower odds of clinically important diarrhea (OR = 0.326, 95% CI [0.115–0.923], *p* = 0.035; OR = 0.186, 95% CI [0.066–0.525], *p* = 0.001, respectively).

#### 3.3.15 Financial difficulties

No additional factors met the bivariate analysis criteria for inclusion; therefore, a final logistic a covariate-only logistic regression model including age, sex, and cancer stage was fitted for financial difficulties.

## 4 Discussion

This study examined HRQoL using the EORTC QLQ-C30 and clinically important impairments and symptoms among cancer patients in Lao PDR. The findings revealed a substantial burden of impaired functioning and symptom distress among cancer patients. More than three-quarters of participants reported clinically important impairments in emotional, physical, cognitive, and social functioning, while financial difficulties, nausea/vomiting, and pain were among the most prevalent concerns. To the best of our knowledge, this is the first study focused exclusively on patients with cancer in Lao PDR to examine HRQoL using the EORTC QLQ-C30 and factors associated with HRQoL within the Lao context. Compared with the overall findings from a multi-country oncology study conducted in eight Southeast Asia countries using the EORTC QLQ-C30, patients in the present study generally reported poorer functioning and greater symptom burden (13). These findings suggest that cancer patients in Lao PDR experience particularly substantial supportive care needs that warrant greater attention in clinical practice and health policy.

In particular, the high prevalence of financial difficulties warrants special attention. Nearly nine out of 10 participants reported clinically important financial problems, indicating that their physical condition or cancer treatment had imposed a substantial financial burden. Although a systematic review of cancer studies using the EORTC QLQ-C30 across Asian countries have consistently identified financial difficulties as one of the most burdensome HRQoL domains (pooled mean score: 39.1), the prevalence of clinically important financial difficulties observed in the present study appears particularly high (20). While cancer-related financial difficulties have been increasingly recognized as a major challenge in LMICs, evidence from Lao PDR remains limited (21, 22). Financial burdens may limit timely access to diagnosis and treatment, reduce healthcare utilization, and contribute to delayed or forgone cancer treatment (22). Consistent with this observation, approximately one-quarter of participants (26%) in the present study had not received any cancer treatment and 82% of untreated patients had stage III or IV cancer. These findings underscore the need for further research on financial difficulties among cancer patients in Laos, as well as interventions to strengthen financial protection and supportive care services, thereby improving HRQoL among cancer patients in Lao PDR.

This study examined the associations between socioeconomic factors and HRQoL. Higher educational attainment was associated with better GQoL. In this study, higher educational attainment was associated with better GQoL. Individuals with higher educational attainment may benefit from greater health literacy, which has been associated with improved engagement in cancer care, contributing to better overall quality of life (23, 24). Individuals with higher educational attainment also tend to have greater socioeconomic resources and better access to healthcare, which may contribute to improved health outcomes and overall quality of life (24). This interpretation is also supported by the finding that only 5% of participants with a bachelor’s degree or higher had not received cancer treatment, compared with 20% of those with a primary or high school education in this study. In addition, participants with higher educational attainment were more likely to report impaired role functioning. One possible explanation is that individuals with higher educational attainment may engage in a wider range of occupational, household, leisure, and social activities. Consequently, cancer-related limitations may have a greater perceived impact on their ability to perform their usual roles and daily activities. Previous studies have shown that cancer and its treatment can reduce participation in work, household activities, usual daily activities, and leisure activities, all of which contribute to role functioning (25). In addition, household income was significantly associated with several HRQoL outcomes; however, income showed mixed and partly counterintuitive associations with HRQoL and should therefore be interpreted cautiously. In particular, household size and local income benchmarks were not available, limiting interpretation of the socioeconomic meaning of household income. Future studies should include more detailed socioeconomic indicators, such as household size, income per capita, and out-of-pocket healthcare expenditures, to better clarify these associations.

Regarding the clinical factors and HRQoL, a notable finding of the study is that participants who had not received treatment generally reported greater symptom burden and poorer functioning than those receiving single-modality or combination therapy. The patients who had not received treatment before or at the time of the survey may include individuals experiencing delayed treatment initiation, limited access to care, or unmet supportive care needs. Despite the growing burden of cancer, oncology services in Lao PDR may remain limited compared with those in neighboring countries. Access to specialized oncology services is constrained by limited infrastructure, diagnostic capacity, essential medications, and shortages of trained healthcare professionals (16, 26–28). In addition to limitations in oncology services, health system challenges, including weak health information systems, financial hardship, limited provider training, and low patient health literacy, may hinder timely cancer diagnosis and treatment (10, 17, 29–31). The findings of the study suggest that improving access to cancer treatment may support symptom control and improved wellbeing and highlight the importance of strengthening access to timely cancer diagnosis, treatment, and supportive services in Lao PDR.

In addition, the present study demonstrated a consistent association between comorbid conditions and poorer HRQoL outcomes. Participants with comorbidities were more likely to report impaired role, emotional, and cognitive functioning, as well as clinically important pain, fatigue, dyspnea, constipation, and diarrhea. This finding is consistent with previous studies showing that multimorbidity increases symptom burden, complicates cancer treatment, and adversely affects quality of life among individuals with cancer (32, 33). The findings highlight the importance of comprehensive symptom management and supportive care for patients with comorbid conditions.

From a public health policy perspective, these findings suggest several plausible priorities for patient-centered cancer care in Lao PDR. Under resource constraints, the high prevalence of financial difficulties, nausea/vomiting, pain, fatigue, and impairments in emotional, physical, cognitive, and social functioning indicates that financial protection, symptom management, supportive care, and palliative care may warrant particular attention. Brief screening could help identify patients with high symptom burden, functional impairment, or financial distress, particularly those with advanced-stage disease, comorbid conditions, or delayed treatment initiation. These patients could then be referred, where available, to symptom management, supportive care, palliative care, psychosocial support, or insurance- and financial-assistance services. Although these policy implications should be interpreted cautiously given the cross-sectional and hospital-based design, the findings provide preliminary evidence to support more systematic assessment of patient needs within existing cancer-care and financial-protection structures in Lao PDR.

Several limitations should be considered. First, the cross-sectional design precludes conclusions regarding causality. Second, participants were recruited from hospitals in Vientiane, the capital city of Laos, where cancer diagnosis and treatment are concentrated. As a result, the findings may not be generalizable to patients living in rural areas or those who do not access hospital-based care. Third, the study did not include several potential confounding sociodemographic variables, such as health insurance status, place of residence (e.g., urban, rural), which may be associated with HRQoL. Finally, several dichotomized outcomes had relatively small numbers of participants in one category, which may have contributed to imprecise estimates and wide confidence intervals. In addition, multiple statistical comparisons were conducted without formal adjustment for multiplicity because the analyses were exploratory. Therefore, the reported *p* values are nominal, and isolated statistically significant findings should be interpreted with caution and not regarded as definitive evidence of an association. Despite these limitations, this study provides important evidence regarding the burden of clinically important impairments and symptoms among cancer patients in Laos and identifies factors that may inform future interventions and supportive care strategies.

Patients with cancer in Lao PDR experienced a substantial burden of impaired functioning, symptom distress, and financial difficulties. Poorer HRQoL was particularly evident among patients who had not received cancer treatment and those with comorbid conditions, while higher educational attainment was associated with better GQoL. These findings highlight the need to improve timely access to cancer diagnosis and treatment, strengthen symptom management and supportive care, and enhance financial protection for patients with cancer. Further research is needed to better understand financial toxicity and barriers to cancer care and to guide interventions aimed at improving HRQoL in Lao PDR.

## Data Availability

The original contributions presented in the study are included in the article/Supplementary material, further inquiries can be directed to the corresponding author.
