# Supplementary material for: Health-related quality of life and associated factors among individuals with cancer in Laos
Source: Front Public Health. 2026 Oct 9;14:1941268. doi: 10.3389/fpubh.2026.1941268 (PMC13690736; doi:10.3389/fpubh.2026.1941268)
Supplement: Supplementary file 1 [file Table_1.docx]

**Supplementary Table 1. Multiple linear/logistic regression analyses for factors associated with health-related quality of life**

| **Variables** | **GQoL (n=184)** | | **Physical functioning (n=184)** | | **Role functioning (n=180)** | | **Emotional functioning (n=184)** | | **Cognitive functioning (n=184)** | |
| --- | --- | --- | --- | --- | --- | --- | --- | --- | --- | --- |
|  | **B (SE)** | **(95% CI)** | **B (SE)** | **OR (95% CI)** | **B (SE)** | **OR (95% CI)** | **B (SE)** | **OR (95% CI)** | **B (SE)** | **OR (95% CI)** |
| Age | **-.246 (.116)** | **(-.476, -.017)^*^** | .038 (.024) | 1.039 (.991, 1.089) | .024 (.018) | 1.025 (.989, 1.062) | .002 (.026) | 1.002 (.952, 1.053) | .016 (.022) | 1.016 (.973, 1.061) |
| Sex: female (ref. male) | .671 (2.828) | (-4.912, 6.253) | .057 (.561) | 1.059 (.352, 3.180) | -.312 (.376) | .732 (.350, 1.531) | -.032 (.659) | .969 (.266, 3.528) | .126 (.479) | 1.134 (.444, 2.900) |
| Marital status: married  (ref. single) | - | - | - | - | - | - | - | - | 1.326 (.861) | 3.766 (.696, 20.374) |
| Education: ≥ Bachelor  (ref. primary/high) | **12.156 (3.019)** | **(6.197, 18.114)^***^** | - | - | **1.217 (.438)** | **3.378 (1.430, 7.976)^**^** | - | - | - | - |
| Household income (per doubling)^a^ | **-9.662 (2.563)** | **(-14.719, -4.535)^***^** | - | - | .531 (.354) | 1.700 (.851, 3.398) | - | - | - | - |
| Comorbid conditions: yes (ref. no) | - | - | .985 (.687) | 2.679 (.697, 10.304) | **.993 (.399)** | **2.700 (1.234, 5.905)^*^** | **1.716 (.834)** | **5.564 (1.085, 28.527)^*^** | **1.513 (.541)** | **4.542 (1.573, 13.114)^**^** |
| Treatment (ref. no) |  |  |  |  |  |  |  |  |  |  |
| Single | 3.370 (3.276) | (-3.096, 9.836) | -.464 (.645) | .629 (.178, 2.226) | -.716 (.464) | .489 (.197, 1.213) | - | - | -.510 (.637) | .600 (.172, 2.092) |
| Combination | **9.252 (3.387)** | **(2.568, 15.937)^**^** | 1.388 (.921) | 4.008 (.659, 24.353) | -.701 (.473) | .496 (.196, 1.254) | - | - | **-1.285 (.614)** | **.277 (.083, .922)^*^** |
| Cancer stage: stage III/ IV (ref. stage I/ II) | **-19.224 (3.041)** | **(-25.227, -13.221)^***^** | .079 (.602) | 1.082 (.333, 3.519) | **.986 (.435)** | **2.681 (1.142, 6.294)^*^** | .314 (.660) | 1.369 (.376, 4.989) | .104 (.498) | 1.109 (.418, 2.944) |
| **Variables** | **Social functioning (n=184)** | | **Pain (n=184)** | | **Fatigue (n=184)** | | **Nausea and vomiting (n=180)** | | **Dyspnea (n=180)** | |
|  | **B (SE)** | **OR (95% CI)** | **B (SE)** | **OR (95% CI)** | **B (SE)** | **OR (95% CI)** | **B (SE)** | **OR (95% CI)** | **B (SE)** | **OR (95% CI)** |
| Age | .017 (.016) | 1.018 (.987, 1.050) | -.004 (.018) | .996 (.961, 1.032) | -.025 (.017) | .976 (.944, 1.009) | .013 (.019) | 1.013 (.977, 1.051) | .019 (.019) | 1.019 (.982, 1.059) |
| Sex: female (ref. male) | .039 (.389) | 1.040 (.485, 2.229) | -.238 (.459) | .788 (.321, 1.936) | -.669 (.394) | .512 (.237, 1.109) | -.016 (.468) | .984 (.393, 2.461) | .-.160 (.394) | .852 (.393, 1.846) |
| Marital status: married  (ref. single) | - | - | - | - | - | - | - | - | 1.184 (.897) | 3.268 (.563, 18.965) |
| Education: ≥ Bachelor  (ref. primary/high) | - | - | - | - | - | - | .870 (.602) | 2.387 (.734, 7.764) | - | - |
| Household income (per doubling)^a^ | - | - | - | - | - | - | **-.817 (.396)** | **.442 (.200, .960)^*^** | **.896 (.372)** | **2.448 (1.181 5.070)^*^** |
| Comorbid conditions: yes (ref. no) | - | - | **1.230 (.488)** | **3.421 (1.315, 8.900)^*^** | **1.447 (.424)** | **4.252 (1.853, 9.759)^***^** | .825 (.463) | 2.281 (.920, 5.654) | **1.088 (.431)** | **2.968 (1.275, 6.910)^*^** |
| Treatment (ref. no) |  |  |  |  |  |  |  |  |  |  |
| Single | - | - | **-1.391 (.639)** | **.249 (.071, .871)^*^** | **-1.581 (.533)** | **.206 (.072, .585)^**^** | - | - | -1.001 (.542) | .367 (.127, 1.063) |
| Combination | - | - | **-1.312 (.650)** | **.269 (.075, .962)^*^** | **-1.528 (.540)** | **.217 (.075, .626)^**^** | - | - | **-1.488 (.542)** | **.226 (.078, .653)^**^** |
| Cancer stage: stage III/ IV (ref. stage I/ II) | .524 (.413) | 1.689 (.752, 3.792) | **.892 (.432)** | **2.439 (1.047, 5.684)^*^** | .539 (.398) | 1.715 (.786, 3.740) | -.319 (.506) | .727 (.270, 1.958) | .550 (.424) | 1.734 (.755, 3.984) |
| **Variables** | **Sleep disturbance (n=184)** | | **Appetite loss (n=184)** | | **Constipation (n=180)** | | **Diarrhea (n=180)** | | **Financial difficulties (n=184)** | |
|  | **B (SE)** | **OR (95% CI)** | **B (SE)** | **OR (95% CI)** | **B (SE)** | **OR (95% CI)** | **B (SE)** | **OR (95% CI)** | **B (SE)** | **OR (95% CI)** |
| Age | .013 (.015) | 1.013 (.985, 1.042) | **.030 (.014)** | **1.031 (1.003, 1.060)^*^** | -.006 (.021) | .994 (.955, 1.035) | .028 (.018) | 1.029 (.993, 1.066) | -.020 (.022) | .981 (.944, 1.019) |
| Sex: female (ref. male) | .337 (.312) | 1.400 (.760, 2.579) | -.092 (.325) | .912 (.483, 1.725) | .012 (.411) | 1.012 (.452, 2.264) | -.334 (.392) | .716 (.332, 1.544) | .308 (.459) | 1.361 (.554, 3.345) |
| Marital status: married  (ref. single) | .681 (.766) | 1.975 (.440, 8.866) | - | - | - | - | - | - | - | - |
| Education: ≥ Bachelor  (ref. primary/high) | - | - | - | - | **1.314 (.418)** | **3.720 (1.641, 8.434)^**^** | .016 (.448) | 1.016 (.422, 2.444) | - | - |
| Household income (per doubling)^a^ | - | - | - | - | **-.966 (.405)** | **.380 (.172, .842)^*^** | - | - | - | - |
| Comorbid conditions: yes (ref. no) | - | - | - | - | **1.335 (.457)** | **3.800 (1.553, 9.299)^**^** | **.972 (.422)** | **2.642 (1.155, 6.047)^*^** | - | - |
| Treatment (ref. no) |  |  |  |  |  |  |  |  |  |  |
| Single | - | - | - | - | - | - | **-1.121 (.531)** | **.326 (.115, .923)^*^** | - | - |
| Combination | - | - | - | - | - | - | **-1.680 (.528)** | **.186 (.066, .525)^**^** | - | - |
| Cancer stage: stage III/ IV (ref. stage I/ II) | .188 (.356) | 1.206 (.601, 2.423) | -.128 (.364) | .880 (.431, 1.796) | -.106 (.450) | .900 (.373, 2.173) | .518 (.413) | 1.678 (.746, 3.774) | -.746 (.648) | .474 (.133, 1.691) |

Note. CI = confidence interval; GQoL= global health status/quality of Life; OR= odds ratio

Age, sex and cancer stage controlled in all models.

Analytic sample sizes varied across regression models because of missing data in variables included in the respective models. Analytic sample sizes are shown for each regression model. Positive and negative outcome-category counts for the overall study sample are reported in Table 2.

 (–) indicates variables not in the final models.

^a^ Household income was natural log-transformed. Regression coefficients and odds ratios for household income are presented per doubling of income to facilitate interpretation.

^*^p < 0.05; ^**^p < 0.01; ^***^ p < 0.001
