# Supplementary material for: Health-related quality of life and associated factors among individuals with cancer in Laos
Source: Front Public Health. 2026 Oct 9;14:1941268. doi: 10.3389/fpubh.2026.1941268 (PMC13690736; doi:10.3389/fpubh.2026.1941268)
Supplement: Supplementary file 2 [file Table_2.DOCX]

**Table 2. Sensitivity analyses using continuous functioning and symptom scale scores**

| **Variables** | **GQoL (n=184)** | | | **Physical functioning (n=184)** | | | | **Role functioning (n=180)** | | | **Emotional functioning (n=184)** | | | **Cognitive functioning (n=184)** | | |
| --- | --- | --- | --- | --- | --- | --- | --- | --- | --- | --- | --- | --- | --- | --- | --- | --- |
|  | **B (SE)** | **95% CI** | **p- value** | **B (SE)** | **95% CI** | | **p- value** | **B (SE)** | **95% CI** | **p- value** | **B (SE)** | **95% CI** | **p- value** | **B (SE)** | **95% CI** | **p- value** |
| Age | **-.246 (.116)** | **(-.476, -.017)** | **.036** | -.203 (.126) | (-.452, -.046) | | .110 | **-.398 (.186)** | **(-.766, -.030)** | **.034** | .-.101 (.153) | (-.402, .199) | .507 | **-.453 (.157)** | **(-.763, -.143)** | **.004** |
| Sex: female (ref. male) | .671 (2.828) | (-4.912, 6.253) | .813 | 2.704 (2.646) | (-2.518, 7.926) | | .308 | 3.807 (4.095) | (-4.277, 11.891) | .354 | 1.978 (3.260) | (-4.456, 8.412) | .545 | .449 (3.075) | (-5.620. 6.519) | .884 |
| Marital status: married (ref. single) | - | - | - | - | - | | - | - | - | - | - | - | - | -4.699 (7.072) | (-18.655, 9.257) | .507 |
| Education: ≥ Bachelor  (ref. primary/high) | **12.156 (3.019)** | **(6.197, 18.114)** | **< .001** | - | - | | - | -7.600 (4.424) | (-16.333, 1.132) | .088 | - | - | - | - | - | - |
| Household income (per doubling)^a^ | **-9.662 (2.563)** | **(-14.719, -4.535)** | **< .001** | - | - | | - | **-8.487 (3.710)** | **(-15.811, -1.162)** | **.023** | - | - | - | - | - | - |
| Comorbid conditions: yes (ref. no) | - | - | - | **-13.435 (2.939)** | **(-19.235, -7.634)** | | **<.001** | **-12.113 (4.320)** | **(-20.641, -3.586)** | **.006** | -4.135 (3.484) | (-11.009, 2.740) | .237 | **-15.689 (3.414)** | **(-22.426, -8.952)** | **< .001** |
| Treatment (ref. no) |  |  |  |  |  | |  |  |  |  |  |  |  |  |  |  |
| Single | 3.370 (3.276) | (-3.096, 9.836) | .305 | **7.877 (3.351)** | **(1.264, 14.489)** | | **.020** | 6.993 (4.914) | (-2.707, 16.693) | .157 | - | - | - | 5.484 (3.905) | (-2.223, 13.191) | .162 |
| Combination | **9.252 (3.387)** | **(2.568, 15.937)^**^** | **.007** | **7.742 (3.386)** | **(1.061, 14.424)** | | **.023** | 6.092 (5.076) | (-3.928, 16.112) | .232 | - | - | - | **10.618 (3.942)** | **(2.839, 18.397)** | **.008** |
| Cancer stage: stage III/ IV (ref. stage I/ II) | **-19.224 (3.041)** | **(-25.227, -13.221)** | **< .001** | -4.649 (3.027) | (-10.623, 1.325) | | .126 | -6.250 (4.429) | (-14.993, 2.493) | .160 | -2.238 (3.729) | (-9.596, 5.119) | .549 | -2.585 (3.519) | (-9.529, 4.359) | .464 |
| **Variables** | **Social functioning (n=184)** | | | **Pain (n=184)** | | | | **Fatigue (n=184)** | | | **Nausea and vomiting (n=180)** | | | **Dyspnea (n=180)** | | |
|  | **B (SE)** | **95% CI** | **p- value** | **B (SE)** | **95% CI** | | **p- value** | **B (SE)** | **95% CI** | **p- value** | **B (SE)** | **95% CI** | **p- value** | **B (SE)** | **95% CI** | **p- value** |
| Age | -.048 (.179) | (-.401, .304) | .787 | .023 (.180) | (-.332, .378) | | .899 | .086 (.143) | (-.197, .368) | .551 | .160 (.187) | (-.210, .530) | .395 | .397 (.242) | (-.081, .875) | .103 |
| Sex: female (ref. male) | -2.982 (4.195) | (-11.259, 5.295) | .478 | -1.329 (3.769) | (-8.768, 6.110) | | .725 | -2.054 (3.003) | (-7.980, 3.871) | .495 | **-10.984 (4.214)** | **(-19.301. -2.667)** | **.010** | -2.199 (4.891) | (-11.852, 7.453) | .653 |
| Marital status: married (ref. single) | - | - | - | - | - | | - | - | - | - | - | - | - | 14.696 (10.996) | (-7.006, 36.397) | .183 |
| Education: ≥ Bachelor  (ref. primary/high) | - | - | - | - | - | | - | - | - | - | 6.893 (4.563) | (-2.114, 15.900) | .133 | - | - | - |
| Household income (per doubling)^a^ | - | - | - | - | - | | - | - | - | - | -3.977 (3.721) | (-11.322, 3.367) | .287 | 4.867 (4.470) | (-3.955, 13.690) | .278 |
| Comorbid conditions: yes (ref. no) | - | - | - | **15.742 (4.187)** | **(7.479, 24.005)** | | **< .001** | **7.814 (3.335)** | **(1.231. 14.396)** | **.020** | **8.686 (4.266)** | **(.265, 17.107)** | **.043** | **14.746 (5.262)** | **(4.361, 25.131)** | **.006** |
| Treatment (ref. no) |  |  |  |  |  | |  |  |  |  |  |  |  |  |  |  |
| Single | - | - | - | **-10.056 (4.774)** | **(-19.477, -.636)** | | **.037** | **-10.430 (3.803)** | **(-17.934, -2.926)** | **.007** | - | - | - | -3.106 (6.037) | (-15.019, 8.808) | .608 |
| Combination | - | - | - | **-12.991 (4.823)** | **(-22.509, -3.472)** | | **.008** | **-11.292 (3.842)** | **(-18.874, -3.709)** | **.004** | - | - | - | -7.228 (5.418) | (-19.567, 5.110) | .249 |
| Cancer stage: stage III/ IV (ref. stage I/ II) | -7.226 (4.784) | (-16.666, 2.214) | .133 | 7.295 (4.312) | (-1.216, 15.805) | | .093 | 4.682 (3.435) | (-2.097, 11.461) | .175 | 6.207 (4.506) | (-2.688, 15.102) | .170 | 4.272 (5.418) | (-6.421, 14.966) | .431 |
| **Variables** | **Sleep disturbance (n=184)** | | | **Appetite loss (n=184)** | | | | **Constipation (n=180)** | | | **Diarrhea (n=180)** | | | **Financial difficulties (n=184)** | | |
|  | **B (SE)** | **95% CI** | **p- value** | **B (SE)** | **95% CI** | **p- value** | | **B (SE)** | **95% CI** | **p- value** | **B (SE)** | **95% CI** | **p- value** | **B (SE)** | **95% CI** | **p- value** |
| Age | .236 (.213) | (-.185, .656) | .270 | **.482 (.197)** | **(.092, .872)** | **.016** | | -.096 (.224) | (-.538, .347) | .670 | .337 (.249) | (-.155, .829) | .178 | -.375 (.235) | (-.837, .088) | .112 |
| Sex: female (ref. male) | 3.953 (4.572) | (-5.070, 12.976) | .388 | -5.649 (4.630) | (-14.785, 3.487) | .224 | | -5.685 (5.041) | (-15.635, 4.265) | .261 | -10.281 (5.305) | (-20.753, .190) | .054 | **13.023 (5.500)** | **(2.170, 23.877)** | **.019** |
| Marital status: married (ref. single) | 8.381 (10.616) | (-12.568, 29.331) | .431 | - | - | - | | - | - | - | - | - | - | - | - | - |
| Education: ≥ Bachelor  (ref. primary/high) | - | - | - | - | - | - | | **20.084 (5.459)** | **(9.309, 30.858)** | **< .001** | **18.588 (5.912)** | **(6.918, 30.258)** | **.002** | - | - | - |
| Household income (per doubling)^a^ | - | - | - | - | - | - | | -7.788 (4.451) | (-16.574, 0.997) | .082 | - | - | - | - | - | - |
| Comorbid conditions: yes (ref. no) | - | - | - | - | - | - | | **21.306 (5.104)** | **(11.232, 31.380)** | **< .001** | **13.020 (5.778)** | **(1.614, 24.426)** | **.026** | - | - | - |
| Treatment (ref. no) |  |  |  |  |  |  | |  |  |  |  |  |  |  |  |  |
| Single | - | - | - | - | - | - | | - | - | - | -8.219 (6.563) | (-21.174, 4.735) | .212 | - | - | - |
| Combination | - | - | - | - | - | - | | - | - | - | -3.903 (6.625) | (-16.979, 9.174) | .557 | - | - | - |
| Cancer stage: stage III/ IV (ref. stage I/ II) | 1.830 (5.218) | (-8.466, 12.126) | .726 | -1.710 (5.281) | (-12.130, 8.709) | .746 | | 6.155 (5.391) | (-4.486, 16.796) | .255 | **11.751 (5.929)** | **(.048, 23.453)** | **.049** | -.304 (6.273) | (-12.682, 12.074) | .961 |

Note. CI = confidence interval; GQoL= global health status/quality of Life; OR= odds ratio

Age, sex and cancer stage controlled in all models.

GQoL was analyzed as a continuous outcome in the primary analysis; therefore, the GQoL model presented here is unchanged from the primary multiple linear regression model.

 (–) indicates variables not in the final models.

^a^ Household income was natural log-transformed. Regression coefficients for household income are presented per doubling of income to facilitate interpretation.
